# Potential Therapeutic Effects of Short-Chain Fatty Acids on Chronic Pain

**DOI:** 10.2174/1570159X20666220927092016

**Published:** 2023-01-17

**Authors:** Yuanyuan Tang, Juan Du, Hongfeng Wu, Mengyao Wang, Sufang Liu, Feng Tao

**Affiliations:** 1 School of Basic Medical Sciences, Xinxiang Medical University, Xinxiang, Henan, China;; 2 Key Laboratory for Molecular Neurology of Xinxiang, Xinxiang, Henan, China;; 3 Department of Biomedical Sciences, College of Dentistry, Texas A&M University Dallas, Texas, USA

**Keywords:** Short-chain fatty acids, chronic pain, gut microbiome, metabolites, gut-brain communication, intestinal diseases

## Abstract

The intestinal homeostasis maintained by the gut microbiome and relevant metabolites is essential for health, and its disturbance leads to various intestinal or extraintestinal diseases. Recent studies suggest that gut microbiome-derived metabolites short-chain fatty acids (SCFAs) are involved in different neurological disorders (such as chronic pain). SCFAs are produced by bacterial fermentation of dietary fibers in the gut and contribute to multiple host processes, including gastrointestinal regulation, cardiovascular modulation, and neuroendocrine-immune homeostasis. Although SCFAs have been implicated in the modulation of chronic pain, the detailed mechanisms that underlie such roles of SCFAs remain to be further investigated. In this review, we summarize currently available research data regarding SCFAs as a potential therapeutic target for chronic pain treatment and discuss several possible mechanisms by which SCFAs modulate chronic pain.

## 1 INTRODUCTION

Previous studies have demonstrated that gut microbiome and relevant metabolites are involved in different types of pain conditions, including chronic neuropathic pain [1, 2], visceral pain [3-6], chemotherapy-induced pain [7], migraine [8, 9], fibromyalgia [10], temporomandibular joint pain [11], and complex regional pain syndrome [12]. The gut microbiome produces a variety of metabolites, such as small organic acids, bile acids, vitamins, choline metabolites, and lipids. Specifically, the gut microbiome can metabolize complex carbohydrates into small organic acids through fermentative reactions. Most products are short-chain fatty acids (SCFAs), mainly consisting of acetate, propionate, and butyrate.

SCFAs are the major gut microbiome-derived metabolites and the most abundant anions in the colon [13]. It has been reported that SCFAs play important roles in gastrointestinal regulation, cardiovascular modulation, and neuroendocrine-immune homeostasis [13]. Accumulating preclinical and clinical evidence implicates a potential role of SCFAs in different neurological disorders: anorexia nervosa [14], Parkinson’s disease [15], Alzheimer’s disease [16], autism spectrum disorder [17, 18], chronic stress [19], and chronic pain [1, 11].

In this review, we summarize recent studies that focus on investigating SCFAs in pain modulation and discuss potential therapeutic application of SCFAs in treating chronic pain. More importantly, we discuss the underlying mechanisms by which SCFAs could be targeted to develop a novel therapy for chronic pain.

## 2 ASSOCIATION BETWEEN SCFAS AND CHRONIC PAIN

SCFAs are free fatty acids with 1-6 carbons atoms, including formic acid (C1), acetic acid (C2), propionic acid (C3), butyric acid (C4), valeric acid (C5), caproate (C6), of which acetic acid, propionic acid and butyric acid are major SCFAs [20, 21]. SCFAs are produced by gut bacterial fermentation of dietary fibers and rapidly absorbed by epithelium to generate ATP and provide energy for colonocytes [22]. They are transported into the portal circulation and metabolized in hepatocytes [13]. SCFAs are the gut's main metabolites and are considered a key regulator in gut-brain communication [20]. Peripherally generated SCFAs can cross the blood-brain barrier (BBB) to enter the brain *via* monocarboxylate transporters [23], which are abundantly expressed in endothelial cells [24]. A previous study shows that ^14^C-SCFAs are detected in rat brains after carotid artery injection [25]. In human studies, SCFAs are detected in the cerebrospinal fluid at the micromolar level [26] and in brain tissues at the picomolar level [27]. SCFAs play an important role in the integrity of BBB to maintain the homeostasis of the central nervous system (CNS) [28].

In germ-free (GF) mice, the three most abundant SCFAs (acetic acid, propionic acid, and butyric acid) are significantly reduced compared with those in specific pathogen-free (SPF) mice [29]. Previous studies have reported that the GF mice display visceral hypersensitivity [6] and show prolonged migraine-like pain in the nitroglycerin (NTG) model [8], and the pain behaviors can be normalized by fecal microbiota transplantation (FMT) [6, 8]. In addition, pain-related receptors and cytokines are increased in the spinal cord of the GF mice [6]. In GF rats subjected to FMT with feces from an irritable bowel syndrome (IBS) patient, berberine treatment elevates acetate, propionate, and total SCFA concentrations, enriches several SCFA-producing bacteria, thereby inhibiting visceral hypersensitivity [30]. These results suggest that SCFAs may be involved in regulating pain hypersensitivity. Besides SCFA production, the microbiome in the gut can regulate pain through other mechanisms, such as mediating gut-brain crosstalk [13]. In this chapter, we discuss the roles of three main SCFAs in pain modulation.

### 2.1 Role of Butyrate in Pain Modulation

Butyrate is the most studied SCFA in the pain research field. Previous studies have observed that butyrate-producing microorganisms are reduced in IBS patients [31] and that supplement of butyrate relieves abdominal pain in patients with IBS [32] and gastrointestinal disorders [33, 34]. Butyrate enemas at physiologically relevant concentrations dose-dependently decrease visceral sensitivity in healthy volunteers [5]. These studies reveal the correlation between butyrate and pain hypersensitivity, which suggests a possible anti-nociceptive effect of butyrate.

The role of butyrate in pain modulation has been investigated in preclinical studies. Increased circulating butyrate is correlated with pain improvement in obese mice after FMT [2]. Moreover, in an IBS rodent model, intestinal administration of butyrate alleviates visceral hypersensitivity [35, 36]. Interestingly, SCFAs-producing gut bacteria also contribute to the inhibition of pain hypersensitivity, such as *Lachnospiraceae*, a butyrate-producing bacterium, which reduces stress-induced visceral hypersensitivity in a rat model [3]. Oral administration of butyrate also alleviates visceral pain [37], chronic constriction injury (CCI)-induced neuropathic pain [37], epilepsy-related persistent pain [38] and NTG-induced migraine-like pain [39, 40] in rodents. Our study found that resveratrol recovers SCFAs-producing gut bacteria and restores gut SCFAs to alleviate inflammatory temporomandibular joint pain [11]. In patients with rheumatoid arthritis and arthritic mice, SCFAs decrease, and butyrate administration reduces arthritis severity [41].

### 2.2 Role of Propionate in Pain Modulation

A clinical study has reported that propionic acid levels in serum are significantly reduced in patients with fibromyalgia [10]. In an *in vitro* model of inflammation, sodium propionate decreases lipopolysaccharide (LPS)-induced high expression of cyclooxygenase-2 and inducible nitric oxide synthase in a concentration-dependent manner [42]; meanwhile, sodium propionate inhibits paw inflammation and shows an analgesic effect in the second phase of formalin test in carrageenan-induced paw inflammation and superoxide anion-induced inflammatory pain rat models [42]. Moreover, sodium propionate and sodium butyrate administration result in pain relief in an NTG-induced migraine-like pain mouse model [39, 40], and such SCFA treatment re-establishes microbiota composition, reduces the expression of proinflammatory mediators, diminishes histological damage in the trigeminal nerve nucleus, and also mitigates the alteration of intestinal mucosa in the ileum [39, 40]. Compared with butyrate, the role of propionate in pain modulation is less studied. Thus, more studies are needed to demonstrate how propionate is involved in pain modulation and the differences between butyrate and propionate in terms of their effects on pain.

### 2.3 Possible Role of Acetate in Pain Modulation

A few studies have shown that acetate has anti-inflammatory effects [43-46], while direct evidence showing its role in pain modulation is lacking. The levels of acetic and propionate acids decrease remarkably in patients with inflammatory bowel disease compared to healthy individuals [47]. In mice, high acetate abundance at sites of inflammation improves pathogen clearance and facilitates the recovery of immunopathology [43]. In a rat model of LPS-induced neuroinflammation, acetate supplementation exerts potent anti-inflammatory and neuroprotective effects by reducing pro-inflammatory cytokine expression and attenuating neuroglial activation [44, 45]. Anti-inflammatory and anti-oxidative effects of acetate have been reported in an LPS-induced mouse model [46].

Accumulating evidence has supported that SCFAs produce beneficial effects on different types of pain [3, 5, 11, 32-41]. However, the following studies suggest that SCFAs may promote pain hypersensitivity. In an IBS-induced visceral hypersensitivity rat model, butyrate contributes to the development of such pain by regulating enteric glial cell-derived nerve growth factor [48]. In a CCI-induced neuropathic pain mouse model, antibiotic treatment reverses CCI-produced abnormalities and inhibits neuropathic pain, and the antibiotic effect is blocked by SCFA administration [1]. In a dextran sodium sulfate-induced colitis mouse model, SCFA-producing gut bacteria and fecal acetate/butyrate increase [49].

Previous studies indicate that SCFAs are involved in developing chronic pain and may play an opposite role in different pain conditions. More studies are needed to further determine the contribution of SCFAs to chronic pain and reveal relevant mechanisms for SCFAs-mediated pain modulation.

## 3 POTENTIAL MECHANISMS BY WHICH SCFAS MODULATE CHRONIC PAIN

**Fig. (1):**
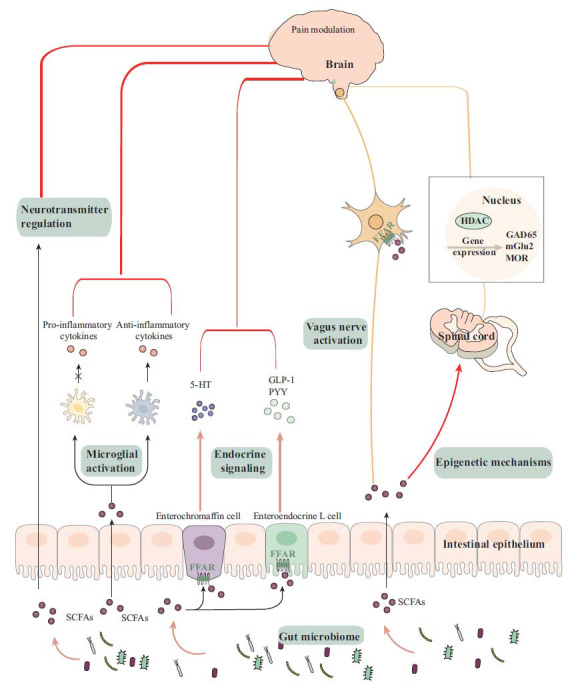
Schematic diagram showing potential mechanisms by which SCFAs modulate chronic pain. SCFAs could contribute to pain modulation through the following mechanisms: histone acetylation-mediated epigenetic mechanism, neurotransmitter regulation, enteroendocrine signaling, vagus nerve activation, and microglial activation. (**Abbreviations:** CNS, central nervous system; FFAR, free fatty acid receptor; GAD65, glutamic acid decarboxylase 65; GLP1, glucagon-like peptide 1; 5-HT, serotonin; mGlu2, metabotropic glutamate receptor 2; MOR, μ-opioid receptor; PYY, peptide YY).

SCFAs exert their effects by binding to SCFA receptors or penetrating cell membranes *via* relevant transporters [50-52]. There are two types of G protein-coupled SCFA receptors (GPR41 and GPR43), which are expressed in both CNS and peripheral nervous system (PNS) [53-55]. GPR41 (also known as free fatty acid receptor 3 (FFAR3)) is a Gi-coupled GPCR [54], and GPR43 (also known as FFAR2) is a Gi/o- and Gq-dual-coupled GPCR [56]. Specifically, GPR41/FFAR3 is expressed in enteroendocrine cells and in ganglion sensory neurons [57, 58] and brain tissues [59]. On the other hand, two major transporters (monocarboxylate transporter (MCT) and sodium-coupled monocarboxylate transporter (SMCT)) mediate the cellular uptake of SCFAs [51, 60]. SCFAs can activate downstream signaling to contribute to different cellular activities through these receptors and transporters. Here, we discuss potential mechanisms that underlie the effects of SCFAs on chronic pain (Fig. **1**).

### 3.1 Epigenetic Mechanisms for SCFAs in Pain Modulation

Histone acetylation is a common epigenetic mechanism for regulating gene expression. *In vitro* studies have demonstrated that SCFAs promote the hyperacetylation of histones by inhibiting the activity of histone deacetylases (HDACs) [61, 62]. HDACs are associated with various neuropsychiatric disorders, including chronic pain [63, 64]. *In vivo* studies further, validate SCFAs-produced HDAC inhibition. For example, sodium butyrate injection (i.p.) results in obvious histone hyperacetylation in mice's hippocampus and frontal cortex [65]. Chronic injection (i.p.) of sodium butyrate for 4 weeks inhibits HDACs, thereby robustly increasing the H3 and H4 acetylation in the hippocampus [66]. Furthermore, intra-hippocampal or intra-medial prefrontal cortex injection of sodium butyrate in mice enhances histone acetylation in different brain regions [67]. Although previous studies in mice have proven that butyrate inhibits HDACs, other SCFAs (acetate and propionate) can also produce inhibition of HDACs to a lesser extent [62, 68].

Taken together, SCFAs are involved in epigenetic mechanisms as an inhibitor of HDACs [61, 62, 65-68], and HDACs have been shown to contribute to pain modulation [69-71]. Specifically, HDAC inhibitors have been reported to produce analgesic effects in chronic pain conditions [37, 38, 72], and previous studies have revealed that SCFA butyrate exerts its analgesic effect in different chronic pain models as a specific HDAC inhibitor [38, 72, 73].

**Fig. (2):**
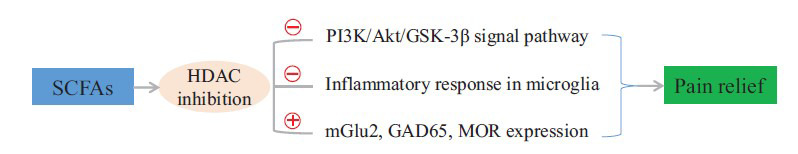
Mechanisms underlying SCFAs-mediated epigenetic regulation of chronic pain. SCFAs function as an inhibitor of HDACs to mediate epigenetic regulation of chronic pain. As shown in the figure, by inhibiting HDACs, SCFAs epigenetically modulate chronic pain through the following mechanisms: 1) inhibiting the PI3K/Akt/GSK-3β signal pathway; 2) diminishing inflammatory response in microglia; 3) up-regulating the expression of mGlu2, GAD65 and MOR in the CNS. Together, SCFAs can produce pain relief. (**Abbreviations:** GAD65, glutamic acid decarboxylase 65; HDAC, histone deacetylase; mGlu2, metabotropic glutamate receptor 2; MOR, μ-opioid receptor; SCFAs, short-chain fatty acids).

Growing evidence indicates that HDACs and histone acetylation regulate pain-related synaptic plasticity, which plays an important role in the development and maintenance of chronic pain [69]. In an animal model of neuropathic pain, HDAC1 expression is upregulated, histone H3 acetylation is reduced in the spinal dorsal horn of rats after spinal nerve ligation, and suppressing spinal HDAC1 expression inhibits neuropathic pain [70]. On the other hand, HDAC2 expression is also increased in rats with neuropathic pain, and inhibition of HDAC2 relieves such pain by inhibiting the PI3K/ Akt/GSK-3β signal pathway [71] (Fig. **2**). These studies suggest that the administration of HDAC inhibitors could be used to treat chronic pain [74]. It has been demonstrated that HDAC inhibition can regulate chronic pain by attenuating inflammatory responses in microglia after peripheral nerve injury [75], modulating the expression of glutamic acid decarboxylase 65 in the brainstem nucleus raphe magnus [76], upregulating metabotropic glutamate receptor 2 and restoring µ-opioid receptors in the spinal cord [77, 78] (Fig. **2**). Moreover, HDAC inhibitors trichostatin A and suberoylanilide hydroxamic acid modulate C-fiber sensitivity by increasing histone acetylation in a nerve injury animal model [79], and these HDAC inhibitors also produce pain relief in a bone cancer pain rat model [77, 80] and enhance morphine analgesia [77]. Combined inhibition of HDACs and Bromodomain and Extra-Terminal domain family proteins could be developed into a novel epigenetic therapy for nerve injury-induced chronic neuropathic pain [81]. Intrathecal injection of baicalin, a nonspecific HDAC inhibitor, ameliorates hyperalgesia and allodynia in the spinal nerve ligation rat model by reversing the expression of H3 acetylation and HDAC1 in the spinal dorsal horn [70]. Valproic acid, another nonspecific HDAC inhibitor, shows a similar inhibitory effect on mechanical allodynia in the neuropathic pain animal model [82].

Besides regulating histone acetylation, SCFAs may also be involved in histone modifications, such as histone crotonylation and hydroxybutyrylation. However, it remains unclear how SCFAs regulate chronic pain *via* these histone modifications. Previous studies have shown a positive correlation among SCFAs, histone crotonylation, and histone acetylation [83, 84], and there are the same dominant sites for crotonylation and acetylation catalyzed by P300 [83]. Butyrate upregulates histone crotonylation, inhibiting physiologically relevant concentrations of HDAC in human colon carcinoma cells and mouse small intestinal organoids [84]. SCFAs may induce histone crotonylation in the CNS, thereby influencing brain function [84]. In addition, β-hydroxybutyrate induces histone β-hydroxybutyrylation instead of acetylation and modulates inflammation in endothelial cells [85].

Therefore, SCFAs, especially butyrate, play a critical role in histone modifications (mainly histone acetylation), which may contribute to epigenetic regulation of chronic pain. However, further studies are needed to provide more evidence for determining the effect of SCFAs on chronic pain *via* different epigenetic mechanisms.

### 3.2 Neurotransmitters Regulated by SCFAs in Pain Modulation

SCFAs regulate the levels of neurotransmitters and neurotrophic factors in the CNS and even produce their effect as a neuromodulator [86]. SCFA treatment decreases nitric oxide, brain-derived neurotrophic factor (BDNF), and neurotrophin-3 in the intestine of mice with NTG-induced migraine [39]. An *in vivo* isotope-labeling study shows that colonic acetate can cross the BBB to reach the brain and increase glutamatergic or GABAergic neurotransmission in the hypothalamus, thereby inducing neuronal activation in the arcuate nucleus [87]. It is well known that glutamatergic and GABAergic neurotransmission systems are involved in the mechanism of chronic pain [88]. Thus, SCFAs may modulate chronic pain through the regulation of these neurotransmitters. Moreover, SCFAs regulate the expression of tryptophan hydroxylase, a key enzyme for serotonin biosynthesis [89], and serotonin has been shown to play an important role in the modulation of chronic pain [90].

SCFAs also regulate neuropeptide levels in the CNS. For instance, SCFAs increase nerve growth factor, glial cell line-derived neurotrophic factor, and BDNF in rat brains, and these neurotrophic factors play critical roles in the physiological activities of neurons and synaptic transmission in the CNS [91-93]. SCFAs may also induce the synthesis of histamine, 5-aminovaleric acid, β-alanine, leptin, peptide YY (PYY), catecholamines, and other neurohormones [86], which are neuroactive compounds involved in the development and maintenance of different types of chronic pain [94-99]. Thus, by regulating neurotransmission and neurotrophic factor levels, SCFAs can promote neuronal activity and contribute to the modulation of chronic pain.

### 3.3 Enteroendocrine Signaling Modulated by SCFAs for Pain Modulation

As mediators of the microbiome-gut-brain axis, SCFAs can modulate the secretion of gut hormones to produce their effect on different physiological activities. It has been reported that SCFAs stimulate the release of glucagon-like peptide 1 (GLP-1) and PYY in the gut of rodents and humans [100, 101]. Increased SCFAs by administering fermentable polysaccharides upregulate circulating levels of GLP-1 and PYY in humans [102]. These gut hormones can activate a signaling cascade that affects different physiological activities through systemic circulation or vagal afferents [20].

GLP-1 is a neuropeptide secreted not only in the gut by enteroendocrine L cells but also in the nucleus tractus solitarius of the brainstem [103]. GLP-1 receptors are expressed in CNS and PNS, influencing brain function *via* different pathways [20]. The circulating level of GLP-1 is related to pain conditions, and decreased GLP-1 in plasma is observed in rats with visceral hypersensitivity [104]. Both circulating GLP-1 and mucosal expression of GLP-1 receptors are negatively correlated with the severity of abdominal pain in IBS patients [105]. Thus, decreased GLP-1 may contribute to pain-related symptoms in abdominal pain patients [106]. GLP-1 and GLP-1 receptor agonists have been shown to reverse pain hypersensitivity. GLP-1 analog ROSE-010 produces pain relief in IBS patients with pain attacks [107]. GLP-1 analog exendin-4 shows a similar effect in a rat model of visceral pain [104]. Subsequent studies have demonstrated the analgesic effect of GLP-1 receptor agonists in various chronic pain rodent models [108, 109]. For instance, GLP-1 receptor agonist exenatide produces an analgesic effect by specifically depleting spinal microglia in a spinal nerve ligation-induced neuropathic pain rat model [109]. Another GLP1 receptor agonist, morroniside, alleviate mechanical allodynia in rats with neuropathic pain *via* GLP-1 receptors/interleukin-10/β-endorphin signal pathway [108].

Like GLP1, anorexic neuropeptide PYY is expressed in many regions of the human brain [110]. PYY crosses the BBB [111] or activates the vagal nerve [112, 113] to regulate brain function in various animal studies [20]. Several studies have shown that PYY and its receptor are involved in the regulation of nociception [98, 114, 115]. A previous study shows reversed balloon distension-induced pain responses, accompanied by increased plasma levels of PYY [114], using gradual intragastric nutrient infusion. Another study indicates that endogenous PYY contributes to somatic thermal and visceral chemical pain and that such an effect is mediated by the Y2 receptor [98] using PYY knockout mice and a Y2 receptor antagonist.

Serotonin (5-HT) is involved in different pain conditions [116]. In the humoral gut-brain communication pathway, serotonin is a key player and can be regulated by SCFAs in the gut. Gut enterochromaffin cells (EECs) synthesize and secret most serotonin [117]. EECs are considered the main sensors to detect changes in the gut lumen, through which they indirectly modulate neural activities [118]. SCFAs promote enteric serotonin production and homeostasis [119]. Intraluminal administration of SCFAs at physiological concentrations promotes serotonin release in rat colon by stimulating EECs [120]. SCFAs acetate and butyrate upregulate the expression of tryptophan hydroxylase 1, the rate-limiting enzyme for mucosal serotonin synthesis, in a human-derived EEC cell model [119]. SCFAs butyrate and propionate, but not acetate, promote serotonin release from RIN14B cells and increase serotonin levels in both colons and serum of GF mice [121].

Additionally, leptin affects nociceptive transmission through specific receptors expressed at the terminal C-fibers or in the trigeminal ganglia [97]. Leptin promotes IL-1 synthesis, which develops chronic pain [122]. SCFAs have modulated leptin production differently; however, the underlying mechanisms are still unclear [123]. Ghrelin can inhibit microglial activation from playing a neuroprotective role in several neuroinflammatory diseases [124-126]. Intra-cerebroventricular or intra-arcuate nucleus injection of ghrelin alleviates formalin-induced pain and increases the level of opioids in the periaqueductal gray area [127]. Ghrelin also produces analgesic effects in animal models of neuropathic pain [128, 129]. A previous study shows that SCFAs decrease plasma ghrelin concentration in rats [130], but more studies are needed to clarify the relationship between SCFAs and ghrelin and its effect on chronic pain.

Furthermore, peroxisome proliferator-activated receptors (PPARs) are members of the nuclear hormone superfamily of receptors, which are activated by fatty acids and their derivatives [131, 132], including SCFAs [133-135]. These nuclear receptors play an important role in glucose, lipid, and lipoprotein metabolism [136-138]. Recently, it has been reported that these receptors modulate both inflammatory and neuropathic pain [131, 138]. PPAR-α and PPAR-γ contribute to the analgesic effect of butyrate on visceral pain and CCI-induced neuropathic pain [37]. In a rat IBS model, butyrate attenuates visceral allodynia and colonic hyperpermeability, which may be PPAR-γ dependent [36]. PPAR-γ is also involved in the analgesic effect of butyrate on epilepsy-related persistent pain [38]. These studies suggest that PPARs could potentially mediate the effect of SCFAs on chronic pain.

### 3.4 Vagus Nerve Activation Induced by SCFAs for Pain Modulation

The vagus nerve is a mixed nerve that contains 80% afferent and 20% efferent fibers and innervates almost all the digestive tract [20]. Vagal afferent fibers in different layers of the gastrointestinal wall, including mucosa, indirectly sense luminal signals of gut hormones and bacterial metabolites (such as SCFAs) [113]. These fibers are the bridge linking gut microbiota and the brain, in which chemoreceptors are activated by gut hormones and SCFAs [118]. The SCFA receptor GPR41/FFAR3 is expressed in vagal neurons [139]. Butyrate induces intracellular Ca^2+^ signaling by acting on nodose ganglion neurons *in vitro* [140]. Administration of *Lactobacillus johnsonii* or *Lactobacillus rhamnosus* enhances gastric vagus nerve activity and regulates the expression of gamma-aminobutyric acid in the brain through the vagal afferent pathway [141, 142]. Supplementation with butyrate in the colon induces an obvious hypotensive effect *via* vagus nerve signaling and SCFA receptors [143]. SCFAs regulate the function of intestinal enterochromaffin cells by acting on vagal afferents, and released serotonin or gut hormones activate respective receptors on vagal afferent fibers [144, 145]. Besides chemoreceptors, vagal afferent terminals can also sense SCFAs (such as butyrate) through direct mechanisms [146].

**Fig. (3):**
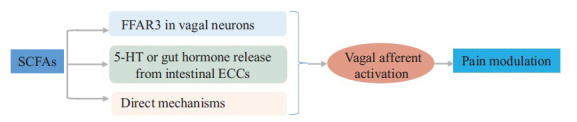
Vagus nerve activation induced by SCFAs for pain modulation. SCFAs can directly or indirectly activate vagal afferents to contribute to pain modulation. SCFA receptor FFAR3 is expressed in the nodose ganglia's vagal sensory neurons, and SCFAs can bind to FFAR3 to produce vagal afferent activation. SCFAs can also promote the release of serotonin or gut hormones from intestinal ECCs. Together, these direct and indirect mechanisms underlie SCFAs-caused vagus nerve activation during chronic pain. (**Abbreviations:** 5-HT, serotonin; ECCs, enterochromaffin cells; FFAR3, free fatty acid receptor 3; SCFAs, short-chain fatty acids).

Besides regulating cardiovascular, respiratory, and alimentary activities, the vagus nerve is also involved in inflammation, mood, and pain modulation [147]. Vagal afferents can be activated by electric stimulation [148] or certain bacterial strains [149] to modulate neurobiological processes in the brain. Previous studies have shown that electrical stimulation of vagal afferents produces an inhibitory effect on spinal nociceptive reflexes and transmission [150-152]. Bacterial metabolites, including lipopolysaccharide, can activate vagal afferents in the gut and nodose ganglia [149]. These studies suggest a role for SCFAs in vagus nerve activation, a potential mechanism for SCFAs-produced pain modulation (Fig. **3**). SCFAs-induced vagus nerve activation could be developed into a novel therapy for chronic pain.

### 3.5 SCFAs-Induced Microglial Activation for Pain Modulation

Accumulating evidence has shown that SCFAs contribute to microglia homeostasis [153] and rearrangement [154]. GF mice display reduced luminal SCFA levels [29], accompanied by SCFA receptor GPR43/FFAR2 deficiency and multiple microglia defects, including altered cell proportions and an immature phenotype [153]. Interestingly, recolonization with complex microbiome or microbiome-derived acetate can restore microglia properties in GF mice [153, 155].

The balance between pro- and anti-inflammatory microglia plays an important role in the pathogenesis of chronic pain. It has been reported that butyrate produces an anti-inflammatory effect in primary brain-derived microglia but produces a pro-inflammatory effect in transformed N9 microglia [156], which suggests that SCFAs can play an opposite role in phenotype polarization of microglia. In the CCI-induced neuropathic pain animal model, there is a positive correlation between SCFA level in the gut and the expression of microglial markers in the hippocampus and spinal cord [1], and SCFA administration regulates microglial activation and subsequent pro-inflammatory phenotype polarization [1]. However, other previous studies [30, 157-160] have shown a beneficial effect of SCFAs on inflammatory diseases. By increasing GPR41/FFAR3 and suppressing the ERK/ JNK/NF-κB pathway, acetate inhibits microglia activation to produce anti-neuroinflammatory effects [157]. In aged mice, butyrate reverses the high expression of pro-inflammatory cytokines in microglia [158]. In addition, berberine treatment promotes SCFAs-producing bacteria growth and reverses IBS-induced visceral hypersensitivity and pro-inflammatory phenotype in microglia [30]. SCFAs improve poststroke neuronal plasticity through circulating lymphocyte-induced microglial activation [159]. Both SCFA mixture and individual SCFA suppress the secretion of pro-inflammatory cytokines (IL-1β, MCP-1, TNF-α, and cytotoxins) *in vitro*, which can be blocked by SCFA receptor antagonism [160].

Moreover, the non-absorbable antibiotic rifaximin promotes the enrichment of *Ruminococcaceae* and *Lachnospiraceae* and increases butyrate levels in the brain, accompanied by the upregulation of anti-inflammatory factors released from microglia in rats [161]. Sesamol, a natural oxidation inhibitor, markedly increases the levels of different SCFAs and significantly downregulates the expression of pro-inflammatory cytokines by suppressing microglial overactivation in the brain of mice with Alzheimer’s disease [162]. Our recent study shows that resveratrol administration reverses the reduction of SCFAs and SCFAs-producing bacteria in the gut and inhibits microglial activation and TNFα expression in the spinal trigeminal nucleus caudalis in an inflammatory temporomandibular joint pain mouse model, thereby alleviating such pain [11].

SCFA deficiency can cause BBB or intestinal barrier deficit, triggering inflammatory reactions by activating microglia. SCFAs decrease in the feces of high-salt diet-fed mice, in which both BBB dysfunction and microglial activation occur, further leading to increased pro-inflammatory cytokine release in the mouse brain [163]. In a dietary fiber deficiency mouse model, gut SCFAs are reduced, and an intestinal barrier is compromised, accompanied by microglial activation and neuroinflammation in the mice [164]. However, a recent study shows that butyrate administration does not significantly alter microglia reactivity and macrophage infiltration in the spinal cord, though the treatment with butyrate diminishes axonal damage and slightly reduces astrocyte reactivity [165].

## CONCLUSION

SCFAs may contribute to the pathogenesis of chronic pain through different mechanisms described in this review. However, more studies are needed to demonstrate how SCFAs modulate nociceptive transmission and chronic pain *via* those mechanisms and whether SCFAs play a critical role in patients with chronic pain. Based on currently available research data in this field, SCFAs could be targeted to develop a novel non-opioid therapy for chronic pain.

## LIST OF ABBREVIATIONS

BBB: Blood-Brain Barrier
CNS: Central Nervous System
FMT: Fecal Microbiota Transplantation
GLP-1: Glucagon-Like Peptide 1
IBS: Irritable Bowel Syndrome
PNS: Peripheral Nervous System
PPARs: Peroxisome Proliferator-Activated Receptors
SCFAs: Short-Chain Fatty Acids
SPF: Specific Pathogen-Free
